# Rasch analysis suggests that health assessment questionnaire II is a generic measure of physical functioning for rheumatic diseases: a cross-sectional study

**DOI:** 10.1186/s12955-018-0939-2

**Published:** 2018-05-30

**Authors:** William J. Taylor, Ketna Parekh

**Affiliations:** 1 Department of Medicine, University of Otago Wellington, PO Box 7343, Wellington, New Zealand; 2 Wellington Regional Rheumatology Unit, Hutt Valley District Health Board, Wellington, New Zealand; 3 General Medicine Service, Capital and Coast District Health Board, Wellington, New Zealand

**Keywords:** Health assessment questionnaire, Psychometric properties, Disability

## Abstract

**Background:**

Versions of the Health Assessment Questionnaire (HAQ) are commonly used to measure physical functioning across multiple rheumatic diseases but there has been no clear demonstration that any HAQ version is actually generic. This study aimed to show that the HAQ-II instrument is invariant across different rheumatic disease categories using the Rasch measurement model, which would confirm that the instrument is generic.

**Methods:**

HAQ-II responses from 882 consecutive rheumatology clinic attendees were fitted to a Rasch model. Invariance across disease was assessed by analysis of variance of residuals implemented in RUMM2030. Rasch modeled HAQ-II scores across disease categories were compared and the mathematical relationship between raw HAQ-II scores and Rasch modeled scores was also determined.

**Results:**

The HAQ-II responses fitted the Rasch model. There was no substantive evidence for lack of invariance by disease category except for a single item (“opening car doors”). Rasch modeled scores could be accurately obtained from raw scores with a cubic formula (R^2^ 0.99). Patients with rheumatoid arthritis had more disability than patients with other kinds of inflammatory arthritis or autoimmune connective tissue disease.

**Conclusions:**

The HAQ-II can be used across different rheumatic diseases and scores can be similarly interpreted from patients with different diseases. Transforming raw scores to Rasch modeled scores enable a strictly linear, interval scale to be used. It remains to be seen how that would affect interpretation of change scores.

**Trial registration:**

ANZCTR ACTRN12617001500347. Registered 24th October 2017 (retrospectively registered).

## Background

According to the World Health Organisation (WHO) International Classification of Functioning, Health and Disability (ICF), the effects of disease or injury are principally manifest as deficits of functioning [1]. Different aspects of functioning have been conceptualized within the ICF model [2]. One aspect of functioning which is intrinsically important to most people with rheumatic disease is ‘activity limitations’. Activity limitations refer to difficulties with day to day activities such as walking, talking, housework or self-care (for example). Activity limitations are typically considered at the individual-level of functioning (that is, without reference to social context). The WHO defines ‘Activity’ as ‘the execution of a task or action by an individual’, which may interact with other components of the ICF model including Environmental Factors that ‘make up the physical, social and attitudinal environment in which people live and conduct their lives’. While activity limitations may be both influenced by and influence social context, for conceptual clarity and measurement, activity limitations are considered separate concepts from social context [3]. One important category of activity limitations concerns physical activities, which is the typical concern of measures of so-called ‘physical functioning’ in the rheumatology literature.

‘Physical functioning’ or ‘disability’ or a similar concept has been endorsed by the Outcome Measures in Rheumatology Clinical Trials (OMERACT) group as a core domain for outcome studies in every rheumatic disease it has considered [4–8]. While there are some disease-specific measures of physical functioning in rheumatology, the most commonly used instrument is the Health Assessment Questionnaire (HAQ) Disability Index and variants [9]. There are several advantages in using the same instrument across different diseases [10]. In particular, direct comparisons can be made with regard to the severity of the functional deficit, which is more difficult when disease-specific instruments are used. It is likely that computer-adaptive testing (CAT) will be even better [11], but in most clinical situations that technology is not easily available [12].

In addition, versions of HAQ scores are one of the 3 components of the Routine Assessment of Patient Index Data 3 (RAPID3) [13] or Patient Activity Scale (PAS, PAS-II) [14] which can be a useful monitor of health status in the clinic situation. The other two components are pain and global assessment of health status/disease activity. Treatment targets and thresholds for low disease activity or remission have been identified for these indices in rheumatoid arthritis. Since the three components of these indices are potentially applicable to any disease where pain and functional deficit are key manifestations, it is possible that they may be generic [15]. For this to be the case, it would be helpful to confirm that the HAQ instrument is also generic. We chose to evaluate the HAQ-II variant of HAQ since it is shorter than the original HAQ-DI (10 items versus 20 items) and was developed using Rasch methodology, which may imply better psychometric properties.

The objective of this study was to demonstrate, using the Rasch measurement model [16], that the HAQ-II instrument was invariant across disease categories. That is, people with different diseases answer the items in the same way (dependent only on their level of function) so that scores can be interpreted in the same way. For example, a score of 2 for a person with rheumatoid arthritis (RA) will mean the same level of disability as a score of 2 for a person with systemic lupus erythematosus (SLE).

## Methods

All patients attending the rheumatology outpatient clinics at the Wellington Regional Rheumatology Unit routinely complete a questionnaire, which consists of the Health Assessment Questionnaire-II (HAQ-II), 10 cm VAS for ‘pain’ and 10 cm VAS for ‘patient global’. The information is mainly used to inform point of care clinical decision making. Data were obtained from 1000 consecutive patient visits over 24 months and were previously reported in an analysis of the PAS-II instrument [15].

The HAQ-II is a 10-item version of the original HAQ-DI, with some new items to extend the range of assessed disability and was derived by fit to a Rasch measurement model [17]. Each item is rated on a 4-point scale (no difficulty, some difficulty, much difficulty, unable to do) and averaged over the number of answered items (must be at least 7) to obtain a total raw score that can range from 0 to 3 (least to most disabled).

The disease diagnoses were divided into 5 diagnostic categories (rheumatoid arthritis (RA), other inflammatory arthritis, auto-immune connective tissue diseases, non-inflammatory disorders and others). “Other inflammatory arthritis” consisted of ankylosing spondylitis, psoriatic arthritis, gout and undifferentiated inflammatory arthritis. “Non-inflammatory disorders” consisted of regional pain syndromes, osteoarthritis and fibromyalgia syndrome. “Autoimmune connective tissue” diseases included SLE, systemic sclerosis and undifferentiated connective tissue diseases. “Others” included conditions such polymyalgic rheumatica, inflammatory myositis, Sjogren’s syndrome, Behcet’s disease, and plantar fasciitis.

Data were fitted to a polytomous unrestricted partial-credit Rasch model using RUMM2030 software [18]. The Rasch model is mathematically expressed below, and essentially means that the probability of any particular response (*x* where *X*_*ni*_ = *x* {0,1, …, *m*_*i*_} associated with the *m*_*i*_ + 1 successive category of item *i*) on any item *i,* is a function of the ‘ability’ (amount of trait, β_*n*_) of the person *n* and the ‘difficulty’ (amount of trait, δ_*i*_) of the item. The thresholds between each of the *m*_*i*_ + 1 categories of each item are denoted by τ_*ki*_ and γ_*ni*_ is a normalizing factor. Some authors claim that only the Rasch model fulfils the axioms of fundamental measurement [19, 20].

$$ \mathit{\Pr}\left\{{X}_{ni}=x\right\}=\mathit{\exp}\left(x\left({\beta}_n-{\delta}_i\right)-\sum \limits_{k=0}^x{\tau}_{ki}\right)/{\gamma}_{ni} $$

Overall model fit was assessed using an item-trait interaction chi-square statistic and Root Mean Square Error of Approximation (RMSEA) [21]. As reported by Tennant and Pallant, large samples (*N* > 500), can lead to statistically significant chi-square tests without substantive misfit in simulated datasets, so we followed the procedure suggested by Tennant and Pallant, by randomly selecting five subsets of 500 participants and fitting these data to the Rasch model independently; and by using the RMSEA index from the whole sample. RMSEA is a model fit index less likely than the chi-square test to be affected by large samples. A value of 0.02 or less was accepted as indicating adequate model fit [21].

Measurement precision was assessed using the Person-Separation-Index (PSI), which can be interpreted in a similar way to Cronbach’s alpha. A PSI of 0.7 means that the score can distinguish between 2 strata of person-ability whereas a value of 0.9 suggests 4 distinct groups of person-ability can be identified [22].

Individual item fit to the Rasch model was assessed with an item-trait interaction chi-square statistic and a normalized item-person interaction fit residual. A Bonferroni-corrected *p*-value of less than 0.05 was taken to indicate misfit for the chi-square test; fit residuals of greater than 2.5 are taken to indicate poor discrimination of the item and fit residuals of less than − 2.5 are taken to indicate excessive good discrimination (overfit). Unidimensionality was assessed by the proportion of independent t-tests of person estimates derived from contrasting sets of items (selected on the basis of positive or negative loading on the first factor of a principal components analysis of residuals) that were significant at the 0.05 level. Where fewer than 5% of t-tests are significant at *p* < 0.05, the data is supportive of unidimensionality [23].

For each item, invariance by disease category was assessed by a 2-way analysis of variance (ANOVA) of the standardized residuals for individuals grouped into 10 classes based on their Rasch-modeled latent trait (physical disability) and the 5 disease categories [24, 25]. A statistically significant F-value for the disease factor indicates a main-effect of disease category on fit to the Rasch model that is independent of the location of the person on the latent trait. This is known as ‘uniform DIF’. A statistically significant F-value for the interaction between disease category and scale location indicates that people with different diseases fit the Rasch model differently depending on where they are on the latent trait. This is known as ‘non-uniform DIF’. A Bonferroni-corrected *p*-value was used to account for multiple hypothesis testing. The sample size calculation for a 2-way ANOVA with 5 categories of disease and 10 classes of scale location is somewhat complex; we used a post-hoc estimation of power in G*Power [26] to detect a medium effect (F = 0.25) with a total sample of 882, given a (conservative) Bonferroni corrected critical *p*-value of 0.0017, 5 categories of disease and 10 classes of scale location. This yielded a power of 84%. There are multiple approaches to determining DIF, but generally different methods have been shown to lead to similar findings [27].

The distribution of HAQ-II scores and the relationship between raw HAQ-II scores and Rasch modelled scores was assessed using SPSS v24. Rasch-modelled scores were re-scaled to be between 0 and 3 (the raw score range) for ease of interpretation by clinicians familiar with HAQ scores. This was accomplished using a linear transformation according to the rescaling formula below, where the range of the Rasch score was observed to be − 5.97 to 4.91 and the range of the rescaled score was 0 to 3.

$$ \frac{{\mathit{\max}}_{rescaled}-{\mathit{\min}}_{rescaled}}{{\mathit{\max}}_{Rasch}-{\mathit{\min}}_{Rasch}}\times \left( value-{\mathit{\max}}_{Rasch}\right)+{\mathit{\max}}_{rescaled} $$

Ethical approval was granted by the New Zealand Health and Disability Ethics Committee without full review as part of its standing procedures for observational, low risk studies. The study was retrospectively registered with the Australian New Zealand Clinical Trials Registry (ACTRN12617001500347).

## Results

From the 1000 consecutive patient visits over 24 months, we selected 882 unique patients with their first visit during the observation period (since some patients visited more than once). About one third of all patient visits had rheumatoid arthritis (RA) (Table 1). Fitting the data to the Rasch model led to an overall chi-square of 122 (df 90), *p* = 0.013 and RMSEA 0.02. The PSI was 0.89, indicating approximately 4 distinct strata of person-ability can be distinguished with the HAQ-II. Unidimensionality was confirmed using the equating t-tests procedure implemented in RUMM (3.78% of t-tests were significant at the 5% level). Each of the five randomly selected subsets of 500 individuals showed overall model chi-square *p*-value > 0.05, confirming that the data fit the Rasch model.

**Table 1:** Participant characteristics

Diagnostic group	N	Percent female	Age, years (mean, SD)
Rheumatoid arthritis	342	72	60.4 (13.5)
Other inflammatory arthritis	304	47	50.1 (14.9)
Non-inflammatory disorder	84	87	56.6 (12.8)
Auto-immune connective tissue disease	125	91	47.8 (16.1)
Other	145	83	59.4 (18.6)

Individual item fit is shown in Table 2. While no item demonstrated evidence of misfit at the Bonferroni-corrected *p*-value, 2 items showed evidence of overfit with fits residuals of less than − 2.5.

**Table 2:** Item location and fit statistics

Item	Location (SE) in logits	Residual	DF	ChiSq (9 df)	*p*-value
1 Getting on and off the toilet	2.30 (0.08)	0.16	681.78	13.67	0.13
2 Open car doors	2.00 (0.08)	0.14	680.89	5.87	0.75
3 Stand up from straight chair	0.89 (0.07)	0.37	684.47	12.27	0.20
4 Walk outdoors on flat ground	0.91 (0.07)	1.78	683.57	13.33	0.15
5 Wait in line for 15 min	−0.25 (0.06)	1.88	675.52	11.62	0.24
6 Reach and get down a 5 lb. object	−0.33 (0.06)	0.11	679.10	6.49	0.69
7 Go up 2 or more flights of stairs	−0.44 (0.06)	−2.67	675.52	18.72	0.03
8 Do outside work	−1.21 (0.06)	− 1.61	676.42	18.22	0.03
9 Lift heavy objects	−1.83 (0.06)	−4.02	687.15	14.99	0.09
10 Move heavy objects	−2.05 (0.06)	− 2.21	683.57	7.26	0.61

**Fig. 1:**
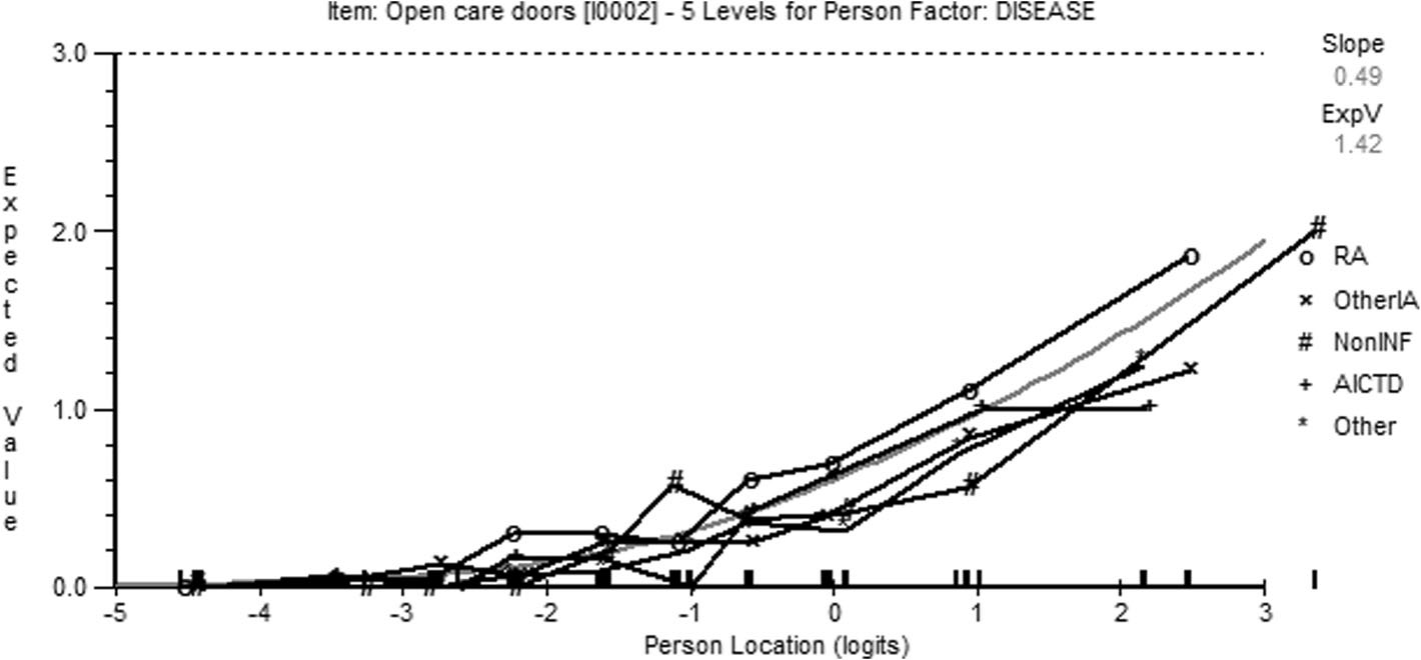
The item-characteristic curve (ICC) for item 2 (opening car doors). This plots the expected response to item 2 based on the individuals’ level of disability (person location). The curves for each disease category are superimposed upon the Rasch model (gray line). DIF would be implied by a significantly different location of a disease-specific ICC. RA (rheumatoid arthritis), IA (inflammatory arthritis), INF (inflammatory disorder), AICTD (autoimmune connective tissue disease)

Differential item functioning analysis is displayed in Table 3. One item (opening car doors) suggested invariance was not present at a *p*-value close to the Bonferroni-corrected level of significance. Inspection of the item-characteristic curve suggested that mostly the ICC for each disease group overlapped, but patients with RA found this item harder than other disease groups, especially for higher levels of disability (to the right of the logit scale) (Fig. 1). However, there was no significant DIF for any item observed in any of the five randomly selected samples of 500 individuals.

**Table 3:** ANOVA for Differential Item Functioning by Disease (item in bold suggests possible DIF at the Bonferroni-corrected level of 0.0017)

Item	Class Interval			Disease			Class Interval x Disease			Total		
	MS	F (df 9)	p	MS	F (df 4)	p	MS	F (df 36)	p	MS	F (df 40)	p
1	1.42	1.61	0.108	0.74	0.84	0.498	1.46	1.65	0.010	55.53	1.573	0.015
**2**	**0.56**	**0.62**	**0.778**	**4.17**	**4.61**	**0.001**	**0.85**	**0.93**	**0.573**	**47.25**	**1.307**	**0.101**
3	1.31	1.42	0.173	1.61	1.75	0.137	0.69	0.74	0.859	31.26	0.849	0.735
4	1.43	1.38	0.192	3.04	2.94	0.019	0.82	0.78	0.809	41.52	1.005	0.465
5	1.5	1.47	0.152	2.6	2.55	0.038	0.54	0.53	0.989	29.88	0.734	0.889
6	0.68	0.75	0.660	1.13	1.25	0.287	0.94	1.03	0.409	38.24	1.06	0.373
7	2.01	2.7	0.004	0.17	0.22	0.926	0.85	1.13	0.269	31.22	1.046	0.395
8	1.91	2.43	0.010	0.65	0.83	0.506	1.06	1.34	0.087	40.76	1.296	0.108
9	1.66	2.38	0.012	2.4	3.43	0.009	0.33	0.47	0.996	21.57	0.773	0.843
10	0.82	1.04	0.402	1.69	2.14	0.074	0.45	0.56	0.981	22.81	0.726	0.896

**Fig. 2:**
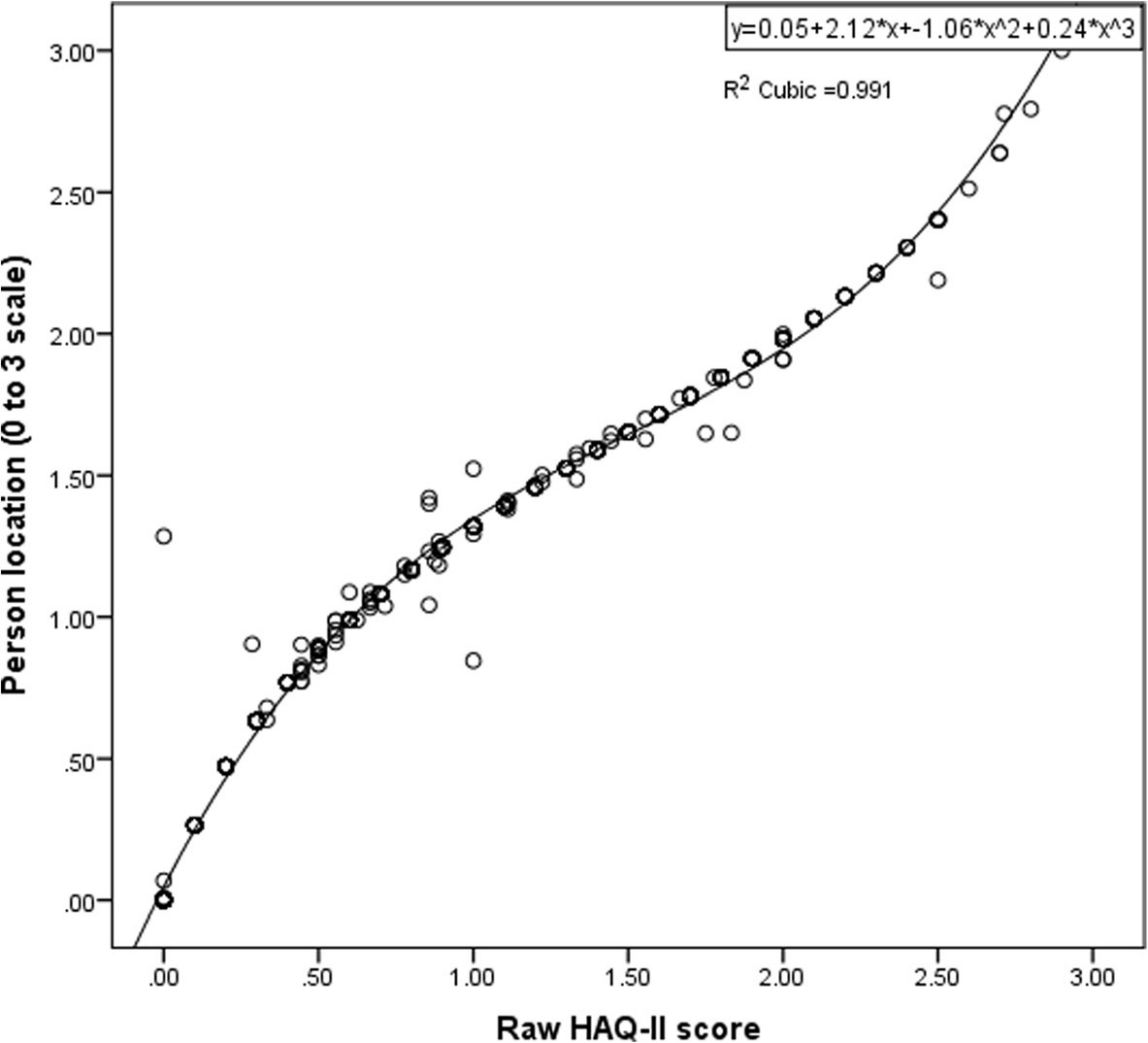
The relationship between Rasch modeled scores and raw HAQ-II scores closely fits a cubic equation

A transformation from a raw HAQ-II score to a Rasch modeled score (rescaled to also range from 0 to 3), but which is now strictly linear, was accomplished by fitting a cubic equation to the relationship between the raw HAQ-II score and the Rasch modeled score (Fig. 2). This equation has an R^2^ of 0.99.

$$ HAQII\ Rasch\ score=0.05+ HAQII\times 2.12-{HAQII}^2\times 1.06+{HAQII}^3\times 0.24 $$

**Fig. 3:**
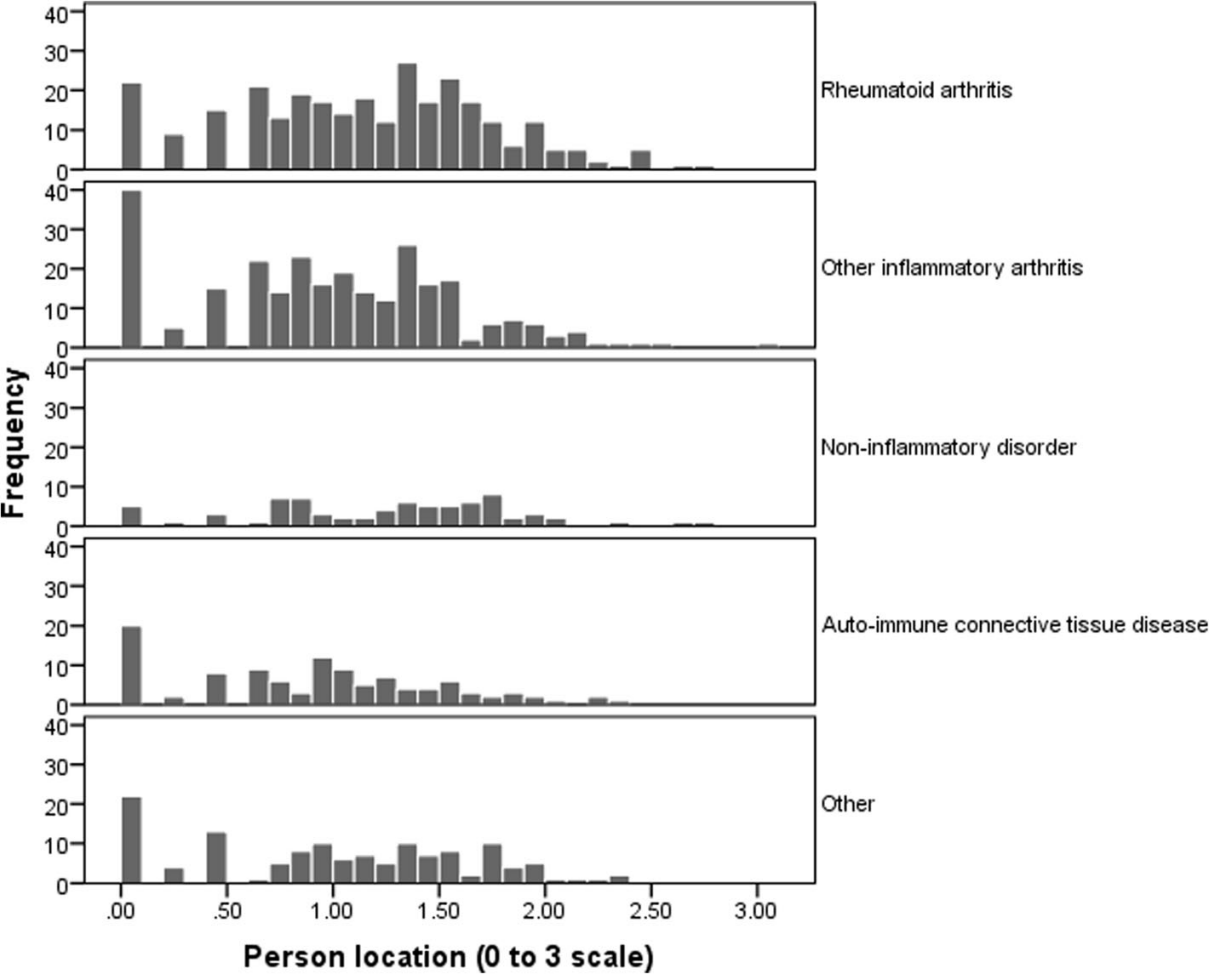
The distribution of Rasch-modeled HAQ-II scores by disease category

The distribution of Rasch modeled scores by disease category is shown in Fig. 3. One way analysis of variance showed that there was a significant difference between the disease categories (F(4,877) = 6.46, *p* < 0.001). Post-hoc tests using RA as the reference disease category showed that RA patients have slightly more disability than patients with other inflammatory arthritis with a mean difference 0.17 (95% CI 0.04 to 0.30, *p* = 0.004) and more disability than patients with autoimmune connective tissue disorders with a mean difference of 0.24 (95% CI 0.07 to 0.42, *p* = 0.002). There were no differences in disability between RA and the other two disease categories.

## Discussion

This study has shown that the HAQ-II instrument can be considered psychometrically generic amongst rheumatology clinic patients. It shows minimal invariance for disease category, which implies that responses to each item and the total score can be interpreted in just the same way for these disease categories. Therefore, it is valid to directly compare physical disability between diseases, and it was found that patients with RA have slightly more disability on average than patients with two other disease categories. The results make the HAQ-II instrument a useful indicator of physical functioning in a general rheumatology clinic, where patients with several different diseases may come for treatment. Furthermore, the HAQ-II instrument can be reasonably incorporated into the PAS-II score for patients with any rheumatic disease to produce meaningful and comparable scores. RAPID3 uses a different version of HAQ, which will require a similar analysis to confirm invariance by disease category.

We have also described a transformation of the raw HAQ-II score that may be useful for aggregated data analysis in audit or clinical research, since it is strictly linear on an interval scale, making it very suitable for parametric statistical analysis and mathematical manipulation.

The meaning of changes in HAQ scores within individuals or between groups is highly dependent upon the linearity of the scale. A non-linear scale makes it very difficult to compare changes at different starting points on the scale, as has been shown for the 10 cm Pain visual analogue scale [28]. The conventional minimal important difference (MCID) for HAQ-DI in RA is 0.20 to 0.22 [29] but may be larger [30]. For HAQ-II, its authors suggest MCID of 0.34. However, MCID assume a linear scale, which is clearly not the case for the raw scores. More meaningful values of MCID should be directly determined using Rasch-modelled scores compared to patient perception of change.

The main limitation of this study is the semi-arbitrary way by which rheumatic diseases were grouped together. It is possible that more distinct diseases may show differential item functioning which is not apparent when two or more diseases are grouped together. On the other hand, grouping similar diseases together may increase the statistical power to show differences, although this assumes that the within-group diseases associate with physical functioning in a similar way. In addition, there is some functional heterogeneity within some relatively defined diseases such as systemic lupus erythematosus and psoriatic arthritis. Overall, it is unclear whether a different approach to grouping diseases would have yielded different results, and could be an avenue for further testing..

## Conclusions

The HAQ-II instrument has good psychometric properties including invariance by disease, suggesting that the measure can be used with confidence in general rheumatology clinics. Although theoretically attractive, it is not yet clear whether transformation of raw scores to a Rasch-modelled score confers practical advantages.

## Abbreviations

ANOVA: Analysis of variance
DIF: Differential item functioning
HAQ: Health Assessment Questionnaire
ICF: International Classification of Health, Functioning and Disability
MCID: Minimal clinically important difference
OMERACT: Outcome Measures in Rheumatology Clinical Trials
PAS: Patient Activity Scale
RA: Rheumatoid arthritis
RAPID3: Routine Assessment of Patient Index Data 3
RMSEA: Root mean square error of approximation
RUMM: Rasch Unidimensional Measurement Models
SLE: Systemic Lupus Erythematosus
SPSS: Statistics Package for the Social Sciences
VAS: Visual Analogue Scale
WHO: World Health Organisation
